# Prevalence of Impaired Bone Health in Premature Ovarian Insufficiency and Early Menopause and the Impact of Time to Diagnosis

**DOI:** 10.3390/jcm14124210

**Published:** 2025-06-13

**Authors:** Szilvia Csehely, Adrienn Kun, Edina Orbán, Tamás Katona, Mónika Orosz, Tünde Herman, Zoárd Tibor Krasznai, Tamás Deli, Attila Jakab

**Affiliations:** 1 Department of Obstetrics and Gynaecology, Faculty of Medicine, University of Debrecen, Nagyerdei krt. 98, 4032 Debrecen, Hungary; kun.adrienn@med.unideb.hu (A.K.); orban.edina@med.unideb.hu (E.O.); orosz.monika@med.unideb.hu (M.O.); krasznai.zoard@med.unideb.hu (Z.T.K.); deli.tamas@med.unideb.hu (T.D.); 2 Doctoral School of Informatics, University of Debrecen, 4028 Debrecen, Hungary; katona.tamas@inf.unideb.hu; 3 Department of Data Science and Visualization, Faculty of Informatics, University of Debrecen, 4028 Debrecen, Hungary; 4 Assisted Reproduction Centre, Clinical Centre, University of Debrecen, Egyetem Tér 1., Nagyerdei krt. 98., 4032 Debrecen, Hungary; herman.tunde@med.unideb.hu

**Keywords:** premature ovarian insufficiency, early menopause, bone health, diagnostic delay

## Abstract

**Background/Objectives:** Premature ovarian insufficiency (POI) is a leading cause of hypoestrogenism in women under the age of 40 years and is associated with an increased risk of impaired bone health. Early diagnosis and timely hormonal intervention are essential to prevent irreversible bone loss. However, diagnostic delay is not uncommon in clinical practice. **Methods:** We conducted a retrospective analysis of 168 women diagnosed with POI or early menopause (EM) between 2017 and 2024 at a tertiary gynecological endocrinology unit. Bone mineral density (BMD) and T-score were assessed by dual-energy X-ray absorptiometry (DXA) at the time of diagnosis in 125 patients, of whom 116 had secondary amenorrhea. The interval between the last menstrual period (LMP) and diagnosis was used to assess the impact of diagnostic delay. The patients were further stratified by serum estradiol (E2) levels and body mass index (BMI). **Results:** At the time of diagnosis, 43.1% of patients had osteopenia, and 10.3% had osteoporosis. A statistically significant negative correlation was observed between time to diagnosis and BMD (r = −0.225, *p* = 0.022), with a similar trend seen for T-score (r = −0.211, *p* = 0.031). In patients with E2 ≤ 5 ng/L, the association was stronger (BMD: r = −0.401, *p* = 0.026). Lower E2 levels tended to be associated with poorer bone health in women with a BMI < 25 kg/m^2^, whereas no such trend was observed in those with a higher BMI. **Conclusions:** Our findings indicate that diagnostic delay in POI is associated with deterioration in bone health, particularly in lean patients and those with severe hypoestrogenism. These results underscore the importance of early recognition and timely initiation of hormone therapy to preserve bone mass and reduce long-term skeletal complications.

## 1. Introduction

POI is a reproductive endocrine disorder characterized by the cessation of ovarian function before the age of 40 years [1].

Estrogen deficiency, which is characteristic of POI, can lead to accelerated bone loss and an increased risk of osteoporosis, since estrogen plays a crucial role in maintaining bone turnover and promoting bone formation [1,2]. Lindsay et al.’s pioneer study highlighted the preventive impact of synthetic estrogen therapy on bone loss in oophorectomized women, underscoring estrogen’s significant role in maintaining bone health [1,3]. Estrogen influences bone metabolism by reducing osteoclast formation, promoting osteoclast apoptosis, inhibiting osteoblast apoptosis, and suppressing sclerostin and oxidative stress products, thereby preventing bone loss and enhancing cortical bone response through ERα activation [4,5,6,7]. Fuller Albright’s groundbreaking work in 1941 was the first to recognize POI and connect estrogen deficiency and menopause with decreased BMD and elevated fracture risk, providing foundational insights into its etiopathogenesis [1,8,9,10].

Osteoporosis presents a significant concern for women diagnosed with POI, with prevalence rates estimated to range between 8% and 27% [11]. Women with POI have significantly lower BMD levels and are at a greater risk of developing osteoporosis compared to women experiencing menopause at the usual age. Specifically, their risk of osteoporosis is approximately 2.54 times higher (with a 95% confidence interval ranging from 1.63 to 3.96), particularly pronounced among women younger than 70 years old [2,11,12]. Several large, well-designed studies have provided strong evidence that menopause occurring at or before the age of 45 years is associated with a 1.5 to 3 times higher fracture risk compared to women who experience natural menopause after the age of 50 years [13,14,15].

Early recognition of POI and proactive management of bone health can help mitigate the risk of osteoporosis and its associated complications in women with this condition [16].

Several factors contribute to the elevated risk of osteoporosis in women with POI, the most relevant of which are discussed below. Individuals experiencing primary amenorrhea are at a heightened risk of developing osteoporosis due to the hypoestrogenic state, which hinders the attainment of optimal peak bone density [1,17]. Peak bone mass (PBM), typically achieved by the age of 18 years in girls and 20 years in boys, is crucial for preventing osteoporosis later in life and is influenced by genetics, gender, and race [1,18]. African-American and Asian ethnicities are identified risk factors for low BMD in spontaneous POI. The duration of the hypoestrogenic state has a profound cumulative impact on bone health. Delayed diagnosis (>1 year) or the onset of irregular menses before the age of 20 years can lead to more significant bone loss [2,19]. Low serum vitamin D levels and inadequate dietary calcium intake also contribute to low BMD [2,11,20]. Unfortunately, patients’ apprehension toward hormone therapy remains common, greatly affecting compliance with estrogen therapy [20,21]. A sedentary lifestyle, low BMI, and a history of smoking also negatively affect bone density [10,11,19].

In Turner syndrome-related POI, bone health is compromised not only by estrogen deficiency but also by factors such as growth hormone deficiency, delayed puberty, and coexisting conditions like inflammatory bowel disease, celiac disease, and liver or thyroid dysfunction, all of which may contribute to decreased BMD and increased fracture risk [11,22].

Autoimmune conditions associated with POI, such as celiac disease, Hashimoto’s thyroiditis, rheumatoid arthritis, hyperthyroidism, type 1 diabetes mellitus, and Addison’s disease, can directly contribute to bone loss. Additionally, long-term glucocorticoid therapy, often required in Addison’s disease, may further exacerbate bone loss [10].

Radiotherapy poses both acute and long-term effects on bone health, potentially leading to disruptions in the bone remodeling process and, in severe cases, conditions like osteoporosis or osteonecrosis. A cumulative dose of approximately 2–4 Gray delivered to both ovaries can result in ovarian damage, potentially leading to POI [4]. Similarly, cytotoxic chemotherapeutic agents can adversely affect bone health and may induce ovarian failure, resulting in a hypoestrogenic state [11].

Consensus among guidelines supports the initiation and continuation of hormone replacement therapy (HRT) until at least the typical age of menopause in cases of POI [2,10,23]. Compared to standard postmenopausal hormone therapy, where the goal is to use the lowest effective dose, patients with POI require higher doses, as the aim is to mimic ovarian function as closely as possible. In early POI with residual ovarian activity and contraceptive needs, continuous use of a combined estrogen–progestin contraceptive pill (COC) may be an alternative option. However, in the long-term, physiological hormone therapy is preferable for maintaining bone health [10,13,23,24,25]. Several comparative studies have examined HRT and COC, demonstrating that estradiol-based HRT is associated with better bone density outcomes [23,24,25,26].

Bone mass is routinely assessed using DXA (GE Medical Systems Lunar, Madison, WI, USA) which provides a two-dimensional measurement of BMD (g/cm^2^) at key skeletal sites, including the lumbar spine (L1–L4), total hip, femoral neck, and distal radius. DXA serves as the gold standard for osteoporosis evaluation and is widely used in clinical practice. According to the World Health Organization (WHO), osteoporosis is diagnosed when BMD falls 2.5 standard deviations below that of a young adult reference population (T-score < −2.5), while osteopenia is defined by a T-score between −2.5 and −1.0 [1,11,27,28]. However, its application in POI patients is more limited, although it remains widely used due to its affordability, non-invasiveness, and low ionizing radiation exposure [11,29].

### Study Objectives

The aim of this study was to investigate the prevalence of osteoporosis among women with POI, particularly in those with primary and secondary amenorrhea, and to assess the impact of early recognition on bone health outcomes. Additionally, we sought to determine the extent of bone density changes associated with different etiological backgrounds of POI.

## 2. Materials and Methods

**Figure 1:**
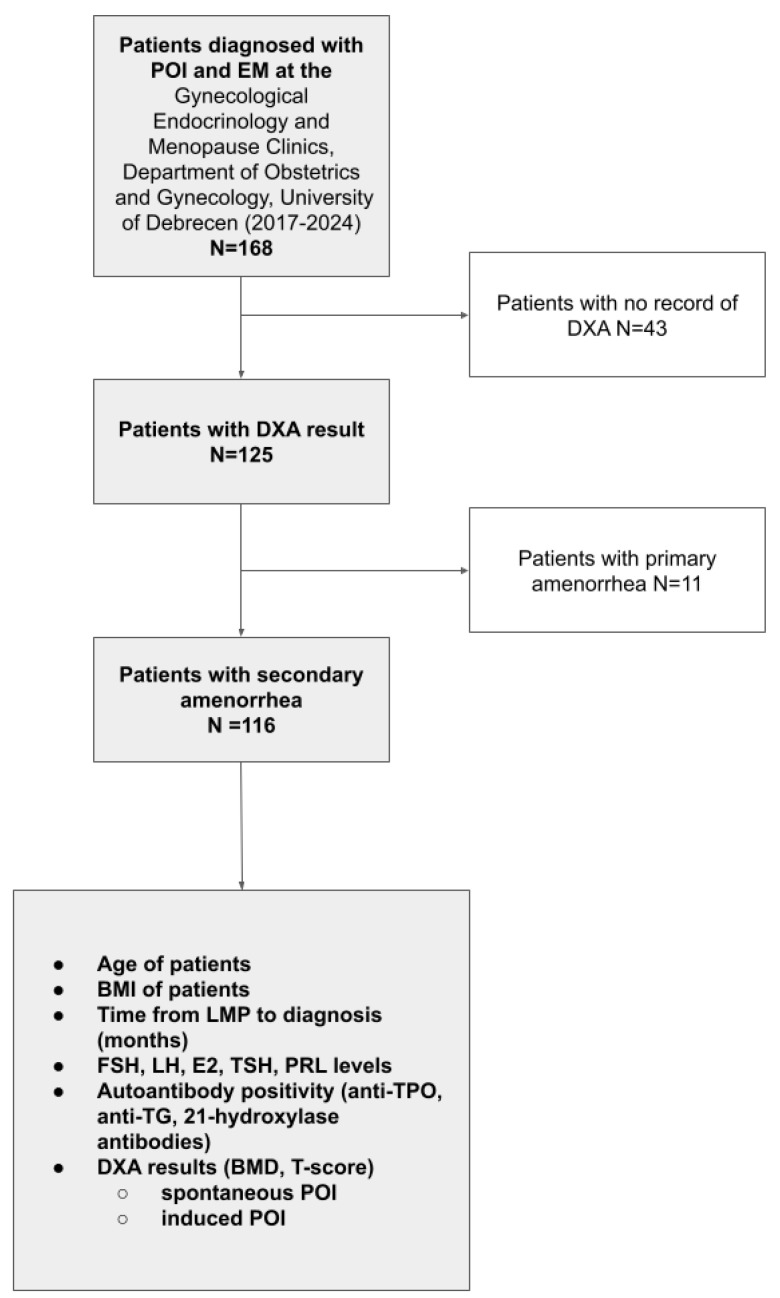
Flowchart of the patient selection for this study.

We conducted a retrospective analysis of 168 consecutively enrolled women (mean age: 36.52 years SD: ±8.89 years) who were diagnosed with primary ovarian insufficiency (POI) or early menopause (EM) at our university hospital-based Gynecological Endocrinology and Menopause Unit between January 2017 and June 2024 and had not received any estrogen and/or progestogen therapy (neither hormone replacement nor hormonal contraception) since their last menstrual period. No formal sample size calculation was performed prior to this study, as all eligible patients during the study period were included. POI diagnosis in the study period was based on established guidelines of the National Institute for Health and Care Excellence (NICE) [30], defined as amenorrhea for at least four months and elevated follicle-stimulating hormone (FSH) level (>30 U/L), confirmed by two separate measurements at least four weeks apart. In accordance with standard definitions, patients under the age of 40 years were classified as having POI, while those aged 40–45 years meeting the same hormonal and clinical criteria were categorized as EM. Patient data were retrospectively retrieved from the electronic medical database system. Detailed medical, gynecologic, surgical, and oncologic history; gravidity; and parity were recorded. According to our standard diagnostic protocol for POI and EM patients, the following data were collected: age at the time of diagnosis, age at the time of last menstrual period, BMI, initial serum FSH, luteinizing hormone (LH), 17β-estradiol (E2), prolactin (PRL), thyroid-stimulating hormone (TSH), triiodothyronine (T3), and thyroxine (T4). To detect autoimmune etiology and the coexistence of autoimmune polyendocrine syndromes (APS), serum anti-thyroid peroxidase (anti-TPO), anti-thyroglobulin antibody (anti-Tg), and adrenal cortex antibody (21-hydroxilase antibody) levels were measured. For identifying genetic etiology, karyotype and FMR1-gene premutation examination were performed. Transvaginal ultrasound (TVUS) was performed to describe uterine, endometrial, ovarian morphology, and follicle count as well as to exclude any minor pelvis pathologies. To assess bone density at baseline, available DXA scan results were retrospectively collected, and a total of 125 patients with accessible data were included in the bone health analysis (Figure 1). Although current recommendations suggest the use of Z-scores for assessing bone health in POI patients, especially in younger individuals who may not have reached PBM, we relied on T-scores due to the availability of existing scan data and the characteristics of the DXA system used, which did not provide Z-scores. This approach also allowed for the inclusion of secondary POI cases and ensured sufficient sample sizes for analysis.

The exclusion criteria included conditions that could cause amenorrhea or osteoporosis due to non-POI-related mechanisms. Patients with central causes of amenorrhea, such as hypothalamic dysfunction (e.g., excessive exercise, low body weight, anorexia nervosa) or pituitary disorders (e.g., hyperprolactinemia, Sheehan’s syndrome), were excluded. Other exclusion criteria included untreated endocrine disorders (e.g., severe thyroid dysfunction, Cushing’s syndrome), chronic diseases affecting bone metabolism (e.g., inflammatory bowel disease, rheumatoid arthritis, chronic kidney disease), long-term glucocorticoid or gonadotropin-releasing hormone (GnRH) agonist therapy, and genetic conditions unrelated to POI (e.g., androgen insensitivity syndrome; 46,XY gonadal dysgenesis).

To evaluate the effectiveness of early recognition, we assessed the time interval expressed in months between the last menstrual period (LMP) and the date of diagnosis, as determined by the first documented elevation in FSH levels. This interval was used to reflect diagnostic efficiency.

Bone densitometry was performed within a month of diagnosis. BMD was measured using a GE Lunar Prodigy Encore (2003) DXA scanner(GE Medical Systems Lunar, Madison, WI, USA). BMD (g/cm^2^) and T-score measurements were obtained at the lumbar spine (L1, L2, L3, L4, and L1–L4) and the left femur. Additionally, the patients’ current BMI (kg/m^2^) was recorded. Bone health was analyzed in relation to the time elapsed from the LMP to diagnosis as well as to E2 levels at diagnosis. The patients were divided into three groups based on their E2 levels: below 5 ng/L, between 5 and 30 ng/L, and above 30 ng/L. Bone health was also analyzed according to different etiological groups.

In terms of etiology, the patients were classified into two main groups: spontaneous (including genetic, autoimmune, or idiopathic) and induced (iatrogenic) POI. If an etiologic factor could be identified, the patients were further subdivided into genetic, autoimmune, and induced (iatrogenic) subgroups. In the absence of a confirmed cause, the etiology was considered idiopathic POI.

The spontaneous POI group included cases of secondary amenorrhea with unknown etiology (idiopathic), familial cases, and autoimmune-related POI. Genetic (chromosomal) cases were analyzed separately as a distinct subgroup, as they primarily involved patients with primary amenorrhea.

The induced POI group consisted of patients with secondary amenorrhea resulting from surgical or oncological treatments. Patients who had undergone extensive surgery, chemotherapy, or radiotherapy for cancer were assigned to the oncological induced origin. Most surgical cases involved procedures performed for endometriosis or ovarian cystectomies/adnexectomies due to ovarian masses that were initially suspected to be borderline or malignant but ultimately proven benign.

The autoimmune group included patients with positive autoantibody tests (e.g., anti-TPO, anti-Tg, or adrenal cortex antibodies), including those diagnosed with Hashimoto’s thyroiditis or Addison’s disease. Patients with other autoimmune disorders, in whom autoantibody production was confirmed and medical therapy was required, were also categorized in this group.

Cases with identified chromosomal abnormalities, such as Turner syndrome or Triple X syndrome, were classified as POI of genetic origin. Secondary POI patients with a positive family history but an unknown origin of POI or EM were considered as unknown origin, although they may represent a distinct entity.

### Statistical Analysis

For our analysis, we used Python 3.12 along with key libraries such as Pandas and NumPy for efficient data manipulation and preprocessing. Statistical modeling was performed using the statsmodels [31] library to ensure robust analytical insights. For data visualization, we leveraged Matplotlib (version 3.9.4) and Seaborn (version 0.13.2) to create clear and informative plots, tailored to highlight key trends and relationships in the data. Additionally, MySQL (version 5.6) was used to store and manage our dataset, enabling seamless access and integration with our analytical workflow. For the statistical analysis, a *t*-test was used for normally distributed groups, while the Mann–Whitney U test was applied for non-normally distributed data. A *p*-value of less than 0.05 was considered indicative of statistical significance. Normality was assessed using the Shapiro–Wilk test. The Pearson correlation coefficient was also used to evaluate linear relationships between continuous variables. In addition, Z-tests and Fisher’s exact tests were applied where appropriate to compare proportions between groups.

## 3. Results

A total of 168 patients attended with POI in the study period: a total of 111 diagnosed with POI and 57 with EM. The mean age was 32.85 ± 8.8 years for the POI patients and 43.67 ± 2.23 years for the EM patients. Seventeen (10.1%) of the POI patients had primary amenorrhea, while 151 patients out of all patients had secondary amenorrhea (94 POI and 57 EM). The average baseline BMI was similar in the POI and EM groups: 25.5 ± 5.81 for POI and 25.23 ± 4.71 for EM. Mean hormone levels were also recorded and compared, with no statistical differences observed between the POI and EM patients. In all POI patients (N = 111), FSH was 83.65 ± 42.25 IU/L, LH was 44.75 ± 18.76 IU/L, and E2 was 10.24 ng/L (IQR: 5–25.29). In all EM patients (N = 57), FSH was 84.79 ± 38.76 IU/L, LH was 44.78 ± 18.81 IU/L, and E2 was 10.91 ng/L (IQR: 6.17–28). Baseline DXA scans were performed in 125 patients within a month after diagnosis. Since the time to diagnosis could not be determined in the cases of primary POI and standardized BMD and T-scores do not exist under the age of 20 years, the final bone health analysis focused on the patients with secondary POI and EM, in whom the relationship between time to diagnosis and bone health could be evaluated. The secondary POI and EM patients together formed the basis of our analysis, as we believe that, although they are treated as two distinct entities in clinical terminology, they represent a clinical continuum in terms of the impact of early diagnosis on preventing long-term diseases. The baseline characteristics, laboratory results, and DXA findings of all patients and subgroups are shown in Table 1.

### 3.1. Time to Diagnosis

**Figure 2:**
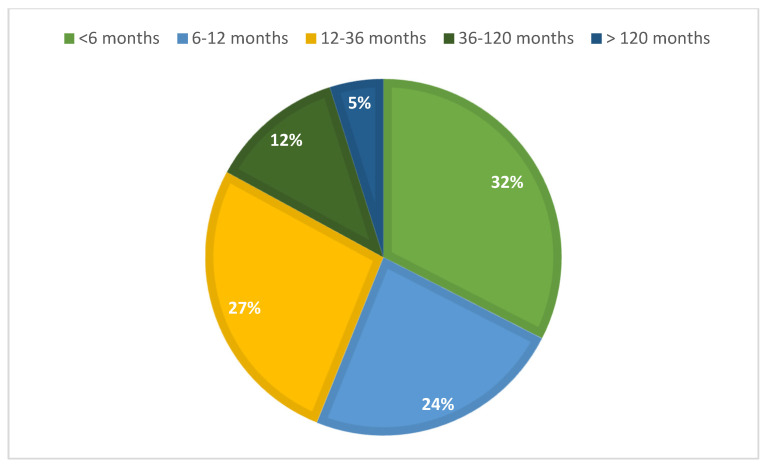
Time to diagnosis in secondary POI + EM patients—distribution across time intervals (N = 116).

The median time elapsed between the last regular period and the diagnosis was 9 months (IQR: 5–23.5) for all POI + EM patients (N = 168), which indicates the low efficiency of early detection. When analyzing only the 116 secondary POI and EM patients with available DXA results, the diagnostic efficiency was distributed as follows. More than one-third of the cases (34%) were diagnosed within the first six months following symptom onset, while an additional 22% received a diagnosis between 6 and 12 months. However, a considerable proportion of patients (27.0%) experienced a diagnostic delay of 1 to 3 years, and 12.0% were diagnosed only after 3 to 10 years. Notably, 5.0% of cases remained undiagnosed for more than a decade (Figure 2).

### 3.2. Baseline DXA Results of All Patients, Primary and Secondary POI, and Etiologic Groups

Mean lumbar and femoral neck BMD and T-score values with standard deviations are shown in Table 1.

**Figure 3:**
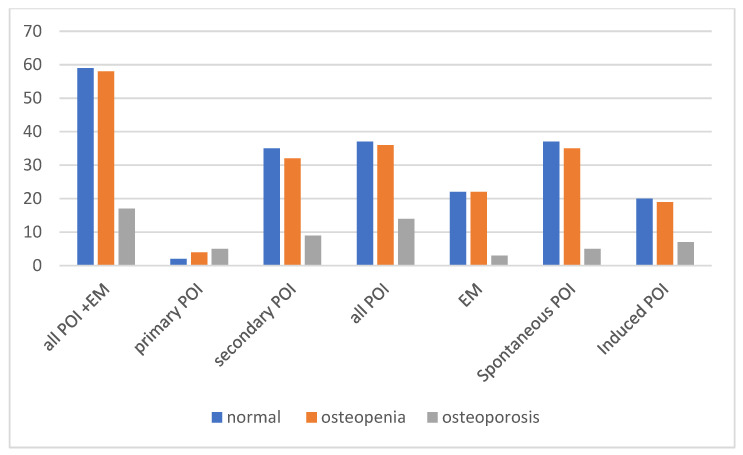
Distribution of normal bone density, osteopenia, and osteoporosis in all POI + EM patients and subgroups (N = 125).

Figure 3 shows the baseline distribution of normal bone density, osteopenia, and osteoporosis among all patients and within the subgroups of primary POI, secondary POI, EM, spontaneous POI + EM, and induced POI + EM. Regarding the clinical diagnoses of bone health, BMD results at the time of diagnosis revealed that impaired bone health (osteopenia or osteoporosis) was present in a substantial proportion of patients across all subgroups. Among all patients with POI and EM, 52 (31.0%) had osteopenia, and 17 (10.1%) had osteoporosis at the time of diagnosis.

The prevalence of osteoporosis was highest among the patients with primary POI (five out of nine; 55.6%) and less among the secondary POI cases, 9 out of 71 (12.7%). Overall, 17.5% (14/80) of the POI patients were affected compared to 6.7% (3/45) in the EM group; however, this difference did not reach statistical significance based on Fisher’s exact test (*p* = 0.108).

When comparing etiologic subgroups, 10 of 50 patients (20%) in the induced POI group had osteoporosis compared to 7 of 75 (9.3%) in the spontaneous POI group; this difference was not statistically significant according to Fisher’s exact test (*p* = 0.112), although the observed trend may warrant further investigation.

Our analysis showed a significant difference in bone health between the induced and spontaneous POI groups diagnosed within the first 12 months based on DXA-based bone categorization (normal vs. non-normal). Of the 20 patients in the induced group (iatrogenic), 14 (70%) were classified as non-normal, whereas 22 (47.8%) of the 46 patients in the spontaneous POI group fell into the non-normal category. A Z-test was performed on the two proportions, which confirmed that the proportion of patients with non-normal bone status was significantly higher in the induced POI group than in the spontaneous group (*p* < 0.05).

### 3.3. Association Between Time to Diagnosis and DXA Results

**Figure 4:**
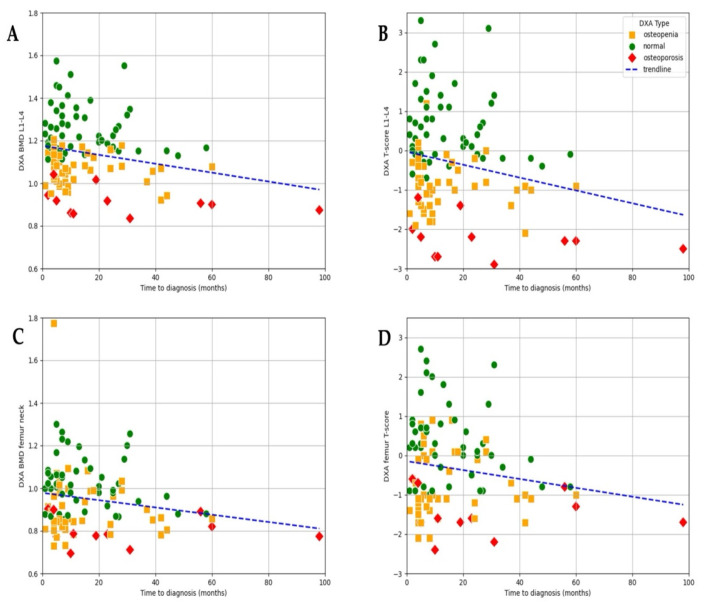
Correlation between time to diagnosis and bone density measurements. (**A**) Lumbar spine BMD (L1–L4) (r = −0.225, *p* = 0.022). (**B**) Lumbar spine T-score (L1–L4) (r = −0.211, *p* = 0.031). (**C**) Femoral neck BMD (r = −0.18, *p* = 0.06). (**D**) Femoral neck T-score (r = −0.17, *p* = 0.08).

At the time of diagnosis, bone health was already compromised in a majority of patients, with 43.1% presenting with osteopenia and 10.3% presenting with osteoporosis, while only 46.6% had normal bone mineral density. A negative trend observed in the scatter plot suggests a clear association between time to diagnosis and lumbar L1–L4 BMD (r = −0.255, *p* = 0.022) as well as T-score (r = −0.21, *p* = 0.031; Figure 4). Although a similar pattern can be observed for femur–neck BMD (r = −0.18, *p* = 0.06) and T-score (r = 0.17, *p* = 0.08), the correlation did not reach significance (Figure 4).

These results indicate that both BMD and T-score may serve as useful indicators of cumulative bone loss related to prolonged estrogen deficiency (Figure 4). Although T-score values showed a wider distribution (range: −2.9 to 3.3; mean −0.299), allowing for the identification of patients with more severe bone loss, the strength of the association with time to diagnosis was only slightly weaker (r = −0.211, *p* = 0.031) compared to that of BMD. The greater variability of T-scores highlights their utility in capturing a broader spectrum of bone health impairment, particularly in advanced cases, while BMD demonstrated a marginally more consistent relationship with diagnostic delay in this dataset.

**Figure 5:**
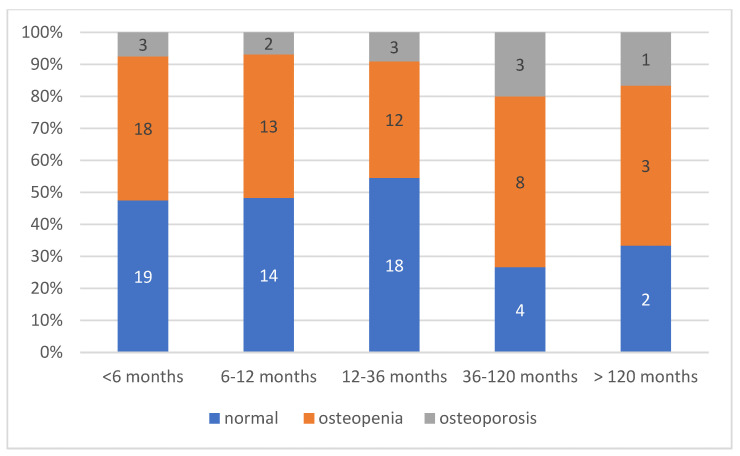
Distribution of normal BMD, osteopenia, and osteoporosis in time-to-diagnosis interval groups (N = 125).

We performed a subgroup comparison to evaluate the distribution of normal bone density, osteopenia, and osteoporosis across different time-to-diagnosis intervals (Figure 5).

The distribution of bone mineral density status in relation to time to diagnosis revealed a clear trend: among the patients diagnosed within 6 months of symptom onset, 18 (45.0%) had normal bone density, 18 (45.0%) had osteopenia, and only 3 (7.5%) were osteoporotic. In contrast, delayed diagnosis was associated with a higher proportion of bone loss. Among the patients diagnosed after more than 3 years (>36 months), the proportion of osteoporosis increased to 25.0% (4 out of 16). However, this difference did not reach statistical significance, although a trend toward increased prevalence of compromised bone health was observed (Figure 5).

### 3.4. Association Between Estradiol Levels, Time to Diagnosis, and DXA Results

**Figure 6:**
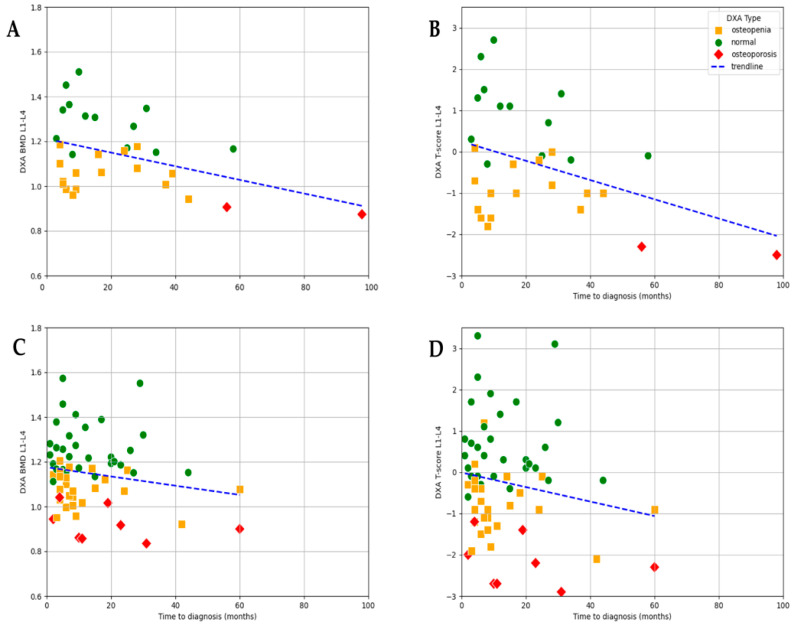
Correlation between time to diagnosis and lumbar bone density measurements according to E2 levels; (**A**) BMD (L1–L4) in patients with E2 ≤ 5 ng/L (r = −0.41, *p* = 0.02); (**B**) T-score (L1–L4) in patients with serum E2 ≤ 5 ng/L (r = −0.38, *p* = 0.036); (**C**) BMD (L1–L4) in patients with E2 > 5 ng/mL (r = −0.17, *p* = 0.18); (**D**) T-score (L1–L4) in patients with serum E2 > 5 ng/L (r = −0.17, *p* = 0.17).

As expected, baseline E2 levels strongly correlated with bone loss. In the subgroup of patients with severe hypoestrogenism (E2 ≤ 5 ng/L), an even stronger statistically significant negative correlation was observed between time to diagnosis and lumbar L1–L4 BMD values (r = −0.401, *p* = 0.026) as well as T-score (r = −0.377, *p* = 0.036). The mean BMD in this group was 1.14 g/cm^2^ (range: 0.874–1.510), and the mean T-score was −0.26 (range −2.5–+2.7), showing considerable variation depending on the timing of diagnosis. This suggests that, among women with profound estrogen deficiency, diagnostic delay may have an even greater impact on bone mineral density (Figure 6).

In the E2 ≤ 5 ng/L subgroup, both BMD and T-score values showed a statistically significant negative correlation with time to diagnosis, with a slightly stronger association observed for BMD (r = −0.41, *p* = 0.026) compared to T-score (r = −0.38, *p* = 0.036; Figure 6).

In the subgroup of patients with higher estradiol levels (E2 > 5 ng/L), no significant correlation was found between time to diagnosis and either BMD or T-score. This aligns with physiological expectations, as the protective effects of estradiol may mitigate the impact of diagnostic delay on bone health in this group (Figure 6).

To further analyze the effect of estrogen levels, the patients were divided into three subgroups based on their baseline E2 levels: ≤5 ng/L, 5–30 ng/L, and >30 ng/L. The results showed that both osteopenia and osteoporosis were more prevalent in the <5 ng/L and 5–30 ng/L groups. Among those with E2 ≤ 5 ng/L, 61.5% had reduced bone density (osteopenia or osteoporosis) compared to 54.5% in the 5–30 ng/L group and 44.4% in the >30 ng/L group. Thus, although a trend toward higher rates of osteopenia and osteoporosis was observed in the patients with lower E2 concentrations, the differences were not statistically significant.

### 3.5. Association Between BMI, Time to Diagnosis, and DXA Results

**Figure 7:**
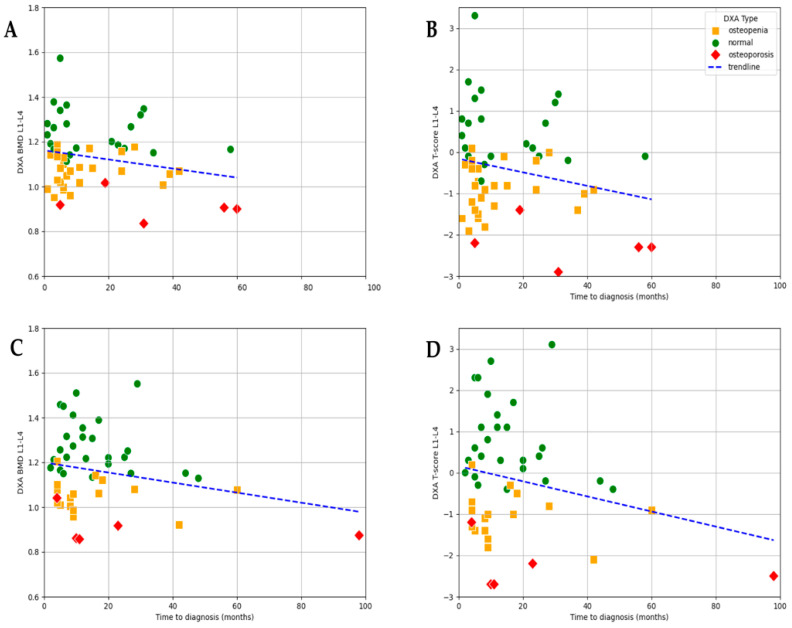
Correlation between time to diagnosis and lumbar bone measurements based on BMI. (**A**) BMD (L1–L4) in patients with BMI ≤ 25 kg/m^2^ (r = −0.225, *p* = 0.109). (**B**) T-score (L1–L4) in patients with BMI ≤ 25 kg/m^2^ (r = −0.217, *p* = 0.12). (**C**) BMD (L1–L4) in patients with BMI > 25 kg/m^2^ 25 (r = −0.23, *p* = 0.11). (**D**) T-score (L1–L4) in patients with BMI > 25 kg/m^2^ (r = −0.23 *p* = 0.12).

In both BMI subgroups, a weak negative correlation was observed between time to diagnosis and total BMD: r = −0.225 (*p* = 0.109) for BMI ≤ 25 kg/m^2^ and r = −0.234 (*p* = 0.113) for BMI > 25 kg/m^2^. When analyzing T-score values in relation to time to diagnosis by BMI subgroups, a weak negative correlation was observed in both groups: r = −0.217 (*p* = 0.121) for patients with BMI ≤ 25 kg/m^2^ and r = −0.230 (*p* = 0.121) for those with BMI > 25 kg/m^2^. Although neither association reached statistical significance, the consistent trend across BMI categories suggests that delayed diagnosis may contribute to declining bone quality irrespective of body mass index (Figure 7).

When comparing femoral BMD values among the patients with a BMI < 25 kg/m^2^ across the different E2 level groups, no significant difference was observed between the E2 ≤ 5 ng/L and E2 ≤ 30 ng/L subgroups (Mann–Whitney U: *p* = 0.459). However, a significant difference was found between the 5–30 ng/L and >30 ng/L groups based on the Mann–Whitney U test (*p* = 0.027), suggesting that femoral bone density may be more preserved at higher estradiol levels, even in lean individuals.

**Figure 8:**
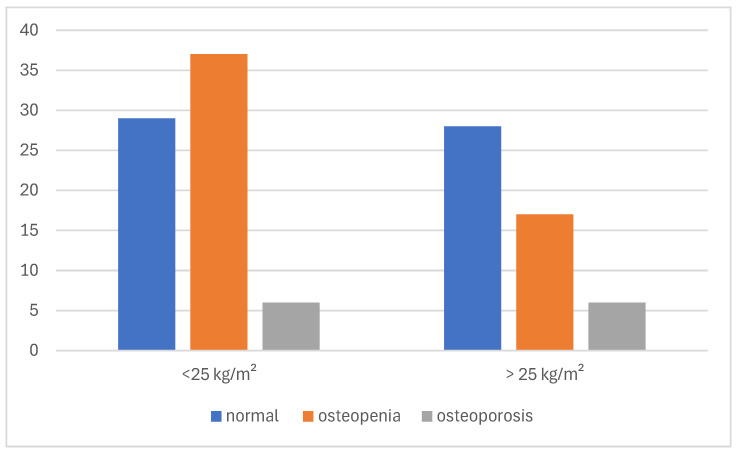
Distribution of normal BMD, osteopenia, and osteoporosis in different BMI interval groups.

Bone health outcomes were also examined in relation to BMI. Among the patients with a BMI ≤ 25 kg/m^2^, 60.3% had either osteopenia or osteoporosis (29 and 10 cases, respectively) compared to 47.2% in those with a BMI > 25 kg/m^2^. Although the difference did not reach statistical significance, a trend toward poorer bone health in leaner patients was observed (Figure 8).

## 4. Discussion

### 4.1. Interpretation of Findings and Clinical Implications

In this study, we assessed the impact of early recognition of POI on bone health. We found that time to diagnosis correlates with bone loss, as expressed by BMD and T-score on the DXA scan. The prevalence of osteopenia and osteoporosis increases with the time to diagnosis of POI in both lumbar spine and femoral neck, although lumbar bone loss appears more sensitive to the duration of estrogen deficiency in the case of POI. Lower estradiol levels and BMI in POI patients increase the risk of faster bone mass decrease; thus, the relatively higher estradiol level in overweight POI patients may provide some protection against rapid bone loss. To our knowledge, this is the first report on the relationship between the time to diagnosis and bone health in POI in which a clear correlation is demonstrated to underline the clinical impact of early diagnosis.

### 4.2. Interpretation of Time to Diagnosis and the Efficiency of Clinical Recognition

More than half of the patients had compromised bone health at the time of diagnosis, which can be attributed to bone loss caused by delayed identification of POI. Our findings indicate that the first three years are crucial for bone metabolism in terms of early recognition. Most diagnoses (56%) occurred within the first year after the onset of amenorrhea; however, the median time to diagnosis remained 9 months (IQR: 5–23.5), suggesting that delayed diagnosis remains a concern in a notable proportion of patients. These observations highlight the importance of timely evaluation and treatment, as late diagnosis may contribute to irreversible reductions in BMD and early fracture risk. Early initiation of HRT could be the key in mitigating these effects and preserving bone health in this population.

### 4.3. Interpretation of Baseline DXA Results in POI Subgroups, EM, and Etiological Categories

Although we expected that early diagnosis would prevent significant bone deterioration, osteoporosis was found to develop early in some cases, depending on the etiology. The patients with primary amenorrhea exhibited the most impaired bone health, which is unsurprising given that an adequate estrogen supply is essential for normal PBM development during adolescence. The attainment of PBM is a highly E2-dependent process, playing a crucial role in determining bone strength and future osteoporotic fracture risk. Since PBM is reached by the end of adolescence, this period represents a critical window for skeletal development [9,17,32].

The failure to achieve PBM during this key developmental stage significantly compromises bone strength and increases the risk of osteoporosis later in life. Furthermore, genetic abnormalities, particularly chromosomal disorders such as Turner syndrome, further contribute to impaired bone health through multiple mechanisms, including intrinsic skeletal dysplasia, estrogen deficiency, and altered growth factor signaling [17,22].

The prevalence of impaired bone health, osteopenia, or osteoporosis tended to be higher in the patients with induced POI based on iatrogenic oncologic or surgical etiology. When comparing BMD between the spontaneous and induced POI patients, a more rapid deterioration in bone metabolism was observed in the induced cases. Median estradiol levels were significantly lower in the patients with induced POI (5.76 ng/L [IQR: 5–14.8]) compared to those with spontaneous POI (15.44 ng/L [IQR: 5–45.7]), as determined by the Mann–Whitney U test (*p* < 0.001). This is likely due to the abrupt decline in E2 levels, which accelerates bone turnover and leads to a more pronounced loss of bone mass compared to spontaneous cases. These results suggest a possible impact of iatrogenic factors on the deterioration of bone health, highlighting the need for closer follow-up and targeted interventions for patients affected by iatrogenic influences.

Interestingly, compromised bone health was less prevalent in the EM group, probably due to the shorter median time to diagnosis (7 months [IQR: 4–18] in EM vs. 14 months [IQR: 7–29.5] in POI, *p* = 0.025) and the fact that these patients had likely already achieved PBM by the time of estrogen decline. We would also speculate that, in cases of amenorrhea, awareness among women of reproductive age or their healthcare providers may be higher than in younger patients, leading to earlier diagnosis.

### 4.4. Associations Between Time to Diagnosis, E2 Levels, and DXA Results

The relationship between the diagnostic delay and lower BMD as well as T-score suggests that women who experience a longer period between the onset of estrogen deficiency and diagnosis tend to have more pronounced bone loss. However, the degree of estrogen deficiency and BMI may modify the dynamics of bone deterioration. Our findings indicate that prolonged and severe estrogen deficiency (E2 ≤ 5 ng/mL) may have a particularly detrimental effect on bone health and that BMD may serve as a useful indicator of bone loss associated with diagnostic delay under conditions of profound hypoestrogenism.

Although there was a negative trend, no significant correlation was observed between time to diagnosis and femoral neck BMD or T-score values. This is in line with existing evidence suggesting that changes in bone density at the femoral neck may occur more gradually, as this site contains predominantly cortical bone, which is less sensitive to early estrogen deficiency. Importantly, bone loss was more prominent in the lumbar vertebrae, likely due to the higher trabecular bone content, which is more metabolically active and sensitive to estrogen deficiency compared to cortical bone, such as that in the femoral neck [6,33,34].

Interestingly, the association between time to diagnosis and bone status appeared to be slightly stronger when using BMD values compared to T-scores in our dataset. This may reflect the fact that BMD measures the absolute mineral content of bone, potentially providing a more direct readout of cumulative estrogen deficiency. In contrast, T-scores are standardized values relative to a young adult reference population and may be more influenced by interindividual variability. While both metrics proved valuable, BMD showed a marginally more consistent relationship with diagnostic delay.

Although the observed correlations were statistically significant, they remained weak with low correlation coefficients (r-values), indicating only modest linear associations. Nevertheless, the presence of significant trends highlights the relevance of diagnostic timing in the context of bone health, even if the strengths of these relationships are limited.

### 4.5. Associations Between BMI, Time to Diagnosis, and DXA Results

In the women with a BMI < 25, lower E2 levels were associated with poorer bone health outcomes, highlighting the stronger impact of estrogen deficiency on bone metabolism in individuals with lower body weight. In contrast, among the women with a BMI ≥ 25, E2 levels did not significantly influence BMD or T-scores. These findings suggest that a higher BMI may partially compensate for the adverse effects of estrogen deficiency on bone metabolism, potentially mitigating bone loss in estrogen-deprived states. This protective effect is likely mediated through increased mechanical loading and peripheral estrogen conversion in adipose tissue. The positive association between BMI and bone mineral density has been confirmed by numerous studies [35,36,37,38]. These findings underscore the importance of early recognition and intervention in estrogen-deficient states to prevent bone loss, particularly in individuals with lower BMI who may be at increased risk.

### 4.6. Closing the Diagnostic Gap in POI Strategies to Enhance POI Detection and Care

Delayed diagnosis of POI can have significant long-term health consequences, particularly regarding bone health. Women who experience a delay of more than one year between the onset of menstrual irregularities and diagnosis are more likely to have lower BMD compared to those diagnosed earlier. Despite increasing awareness, studies have shown that, in a substantial proportion of cases, several years may elapse before POI is recognized, often due to attributing menstrual irregularities to stress or other transient factors. This delay prolongs the period of estrogen deficiency, potentially exacerbating bone loss [19,39]. To address this, it would be essential to increase awareness about POI symptoms among both patients and healthcare providers and improve screening methods or guidelines to facilitate earlier diagnosis.

Menstrual irregularities are often overlooked or not taken seriously, which can lead to delays in diagnosis. In the case of POI, early recognition is particularly crucial, as timely intervention may help mitigate long-term complications, including compromised bone health and an increased risk of osteoporosis. Given that time is a critical factor in the diagnostic process, raising awareness among both healthcare providers and patients is essential to minimize the risk of missed or delayed diagnoses. A thorough evaluation, including personal and family history as well as POI-specific risk factors, should be prioritized. Furthermore, comprehensive clinical and genetic counseling can empower affected women by providing them with the necessary information to explore treatment options and plan for the future [23].

In cases of iatrogenic menopause, particularly in oncology-related POI, improvements can be made by integrating endocrine consultation into oncological treatment protocols. Before ovarian surgery or gonadotoxic therapies, patients should be referred to an endocrinologist as early as possible to address the risk of iatrogenic menopause. Moreover, if the tumor’s hormone receptor status allows for it, hormone therapy should be initiated promptly to help mitigate the adverse effects of estrogen deficiency.

Similarly, early menopause should not be underestimated, as adequate treatment is essential to protect long-term health. Women experiencing early menopause should receive appropriate hormone therapy at least until the average age of natural menopause to reduce the risk of osteoporosis and other long-term complications associated with estrogen deficiency [2,24].

BMD assessment using DXA should be performed at the time of POI diagnosis. The need for follow-up scans should be guided by the initial results, individual risk factors, and adherence to HRT. According to the recommendations of the International Menopause Society (IMS), in women diagnosed with osteoporosis or low bone density, DXA should be reassessed every 1–3 years based on individual risk factors, while those with normal BMD who maintain adequate systemic HRT have a low need for routine reassessment within five years. Further monitoring in these cases should be determined by clinical risk factors [10,24].

### 4.7. Limitations

This study has certain limitations, including its retrospective design, the relatively small sample size, and missing data in some archived records, which may have influenced the completeness and consistency of the dataset. While both BMD and T-score values showed a statistically significant association with diagnostic delay and estrogen deficiency in our analysis, the correlation coefficients remained low. This may be attributed to additional unmeasured factors such as physical activity, vitamin D status, nutritional intake, and genetic predisposition. These findings underscore the need for future studies to employ multivariate models to clarify the relative contribution of these factors and improve predictive risk assessment.

Although DXA is the clinical gold standard for diagnosing osteopenia and osteoporosis and is recommended for young women with prolonged hypoestrogenic amenorrhea, it has notable limitations [1,16]. It does not differentiate between cortical and trabecular bone, does not assess bone quality or geometry, and may require adjustments for short stature, as seen in Turner’s syndrome [1,27,28]. Furthermore, the relationship between BMD and fracture risk remains insufficiently studied in young women with POI, highlighting the need for additional research and complementary diagnostic methods to ensure a more comprehensive evaluation of bone health [1].

Taken together, these limitations weaken the strengths of our conclusions and should be considered when interpreting the results.

## 5. Conclusions

To our knowledge, this is the first study to comprehensively investigate the relationship between diagnostic delay, estrogen deficiency, body composition, and bone health in women with POI and early menopause. While our findings suggest important trends, the observed associations were modest, underscoring the multifactorial nature of bone health of this population. This complex interplay has not been previously addressed in a unified diagnostic and metabolic context. Given the lifelong consequences of impaired bone health and the preventable nature of many of these complications, our findings highlight a critical yet underrecognized area that deserves greater clinical awareness and further research attention.

## Figures and Tables

**Table 1:** Age, BMI, hormone laboratory data, DXA results, and time to diagnosis of all POI patients and subgroups.

	All POI + EM Patients with DXA (N = 125)	Primary POI Patients (N = 9)	All Secondary POI and EM Patients (N = 116)	All POI Patients (N = 80)	EM Patients (N = 45)	Spontaneous POI + EM (N = 75)	Induced POI + EM (N = 50)
**Age (years, ±SD)**	37.92 ± 8.01	**23.55 ± 11.57**	**39.04 ± 6.51**	**34.54 ± 8.08**	**43.95 ± 2.31**	38.37 ± 7.83	37.27 ± 8.3
**BMI (kg/m^2^, ±SD)**	25.31 ± 5.33	**20.79 ± 3.51**	**25.64 ± 5.31**	25.35 ± 5.59	25.21 ± 4.87	24.64 ± 4.35	26.25 ± 6.42
**FSH (IU/L, ±SD)**	84.65 ± 39.86	**105.06 ± 54.11**	**83.07 ± 38.44**	85.34 ± 41.51	83.49 ± 37.33	81.77 ± 38.12	89.78 ± 42.8
**LH (IU/L, ±SD)**	45.46 ± 19.07	49.23 ± 18.45	45.15 ± 19.17	45.77 ± 18.93	44.89 ± 19.55	45.09 ± 17.85	46.15 ± 21.41
**E2 (ng/L, med. IQR)**	10.26 (5–28.95)	**5 (5–8.15)**	**12.16 (5–29.75)**	8.8 (5–25.04)	14.4 (6.23–33.75)	**15.44 (5–45.7)**	**5.76 (5–14.08)**
**TSH (mIU/L, ±SD)**	1.89 ± 1.43	1.88 ± 0.94	1.89 ± 1.46	2.03 ± 1.65	1.66 ± 0.88	1.87 ± 1.11	1.95 ± 1.88
**PRL (** **u** **g/L, ±SD)**	10.23 ± 5.24	11.46 ± 4.73	10.14 ± 5.31	10.82 ± 6.05	9.43 ± 3.89	9.86 ± 3.81	10.91 ± 7.27
**DXA BMD L1–L4 (g/cm^2^, ±SD)**	1.13 ± 0.15	1.02 ± 0.14	1.14 ± 0.15	1.11 ± 0.15	1.16 ± 0.16	1.14 ± 0.15	1.12 ± 0.16
**DXA T-score L1–L4, ±SD**	−0.39 ± 1.28	**−1.38 ± 1.15**	**−0.32 ± 1.26**	−0.53 ± 1.25	−0.16 ± 1.31	−0.33 ± 1.24	−0.48 ± 1.35
**DXA BMD** **femur neck (g/cm^2^, ±SD)**	0.94 ± 0.15	0.88 ± 0.13	0.95 ± 0.15	0.96 ± 0.17	0.91 ± 0.11	0.95 ± 0.16	0.93 ± 0.14
**DXA femur** **T-score, ±SD**	−0.39 ± 1.07	−0.83 ± 1.07	−0.36 ± 1.07	−0.27 ± 1.14	−0.59 ± 0.92	−0.36 ± 1.03	−0.43 ± 1.15
**Time to diagnosis** **(months, median IQR)**	10 (5–26.5)	-	10 (5–26.5)	**14 (7–29.5)**	**7 (4–18)**	8 (5–25.5)	14 (7–27)

Demographic, hormonal, and bone density parameters in women with POI and EM across subgroups with available DXA data. Data are presented as mean ± SD for normally distributed variables and as median (IQR) for non-normally distributed variables (E2 and time to diagnosis). Normality was tested using the Shapiro–Wilk test; in the case of non-normal distribution, the Mann–Whitney U test was applied. Bolded data pairs represent statistically significant differences (*p* < 0.05). Abbreviations: SD—standard deviation, BMI—body mass index, FSH—follicle-stimulating hormone, LH—luteinizing hormone, E2—estradiol, IQR—interquartile range, TSH—thyroid-stimulating hormone, PRL—prolactin, DXA—dual-energy X-ray absorptiometry, BMD—bone mineral density, T-score—SD from young adult mean BMD.

## Data Availability

The data presented in this study are available by contacting the corresponding authors.
